# Integrating GEO, network pharmacology, and in vitro assays to explore the pharmacological mechanism of Bruceae Fructus against laryngeal cancer

**DOI:** 10.1007/s00210-023-02869-9

**Published:** 2023-11-30

**Authors:** Zhongbiao Wu, Zhongyan Zhu, Liyuan Fu

**Affiliations:** Jiangxi Hospital of Integrated Traditional Chinese and Western Medicine, Nanchang, 330003 Jiangxi China

**Keywords:** Network pharmacology, GEO database, Molecular docking, In vitro assays, Bruceae Fructus, Laryngeal cancer

## Abstract

The goal of this study is to look into the pharmacological mechanism of Bruceae Fructus in conjunction with GEO, network pharmacology, and in vitro assays for the treatment of laryngeal cancer to provide theoretical support for its therapeutic use. The active components and matching targets of Bruceae Fructus were retrieved from the TCMSP database, while genes linked with laryngeal cancer were obtained from the GEO, GeneCards, DisGeNET, and DrugBank databases. Besides, the components and targets were supplemented by literatures in PubMed database. Cytoscape software was used to create the active ingredients–target network diagram. The String database was used to build the PPI network. Following that, the core targets were subjected to GO enrichment and KEGG pathway analysis using the DAVID database. Finally, AutoDock was used to perform molecular docking between the core components and the core targets. To investigate the biological effects of beta-sitosterol, the viability of laryngeal cancer cells was assessed after beta-sitosterol therapy using the MTS technique. Following that, how beta-sitosterol affected colony formation after 14 days of culture of treated cells was researched. Flow cytometry was utilized to detect apoptosis to examine the influence of beta-sitosterol on laryngeal cancer cell apoptosis, and then detected mRNA and protein expression levels of 10 key genes by RT-qPCR and Western Blot assay. There were 1258 laryngeal cancer–related genes and 15 Bruceae Fructus components, with beta-sitosterol and luteolin serving as key components. Bruceae Fructus’ primary targets against laryngeal cancer were *IL6*, *JUN*, *TNF*, *IL2*, *IL4*, *IFNG*, *RELA*, *TP53*, *CDKN1A*, and *AKT1*. GO enrichment yielded 41 CC, 78 MF, and 383 BP. Platinum drug resistance, the *PI3K-Akt* signaling pathway, the *p53* signaling pathway, apoptosis, the *HIF-1* signaling pathway, and 147 additional pathways have been added to KEGG. The results of molecular docking revealed that the core components had a high affinity for the core target. The results of the cell experiment indicate that beta-sitosterol suppressed Hep-2 cell activity in a concentration-dependent manner. Besides, beta-sitosterol has powerful antiproliferative properties in Hep-2 cells. Flow cytometry results showed that beta-sitosterol promoted laryngeal cancer cell apoptosis in a concentration-dependent manner. The results of RT-qPCR and Western Blot assay showed that the mRNA and protein expression levels of *TP53*, *JUN*, *TNF-α*, *CDKN1A*, and *IL-2* were significantly up-regulated after beta-sitosterol treatment, while the mRNA and protein expression levels of *RELA*, *AKT1*, *IL-6*, *IFNG*, and *IL-4* were significantly down-regulated. This study integrating GEO, network pharmacology, and in vitro assays investigated the probable mechanism of Bruceae Fructus’ anti-laryngeal cancer activity, which can give a theoretical foundation for additional future animal experiments.

## Introduction

Laryngeal cancer is a malignant tumor of the larynx. Its occurrence ranks second among malignant tumors of the head and neck, with an increasing global trend (Rothman et al. 1980), and its survival rate is similarly dropping (Siegel et al. 2016). The specific pathogenesis of laryngeal cancer is still being researched. At this time, it is known that smoking, drinking, human papilloma virus (HPV) infection, environmental factors, dietary variables, and chronic inflammation all increase the risk of laryngeal cancer. Smoking is connected with the development of throat cancer linearly, with smokers having a risk that is 10 to 15 times that of nonsmokers (Kuper et al. 2002). Additionally, there is a direct correlation between drinking alcohol and the chance of developing laryngeal cancer (Boffetta and Hashibe 2006). Besides, research shows that smoking and drinking alcohol both increase the risk of developing laryngeal cancer (Bosetti et al. 2002). Exposure to harmful environments can also increase the risk of throat cancer, such as asbestos, polycyclic aromatic hydrocarbons (PAHs), engine exhaust, and textile dust (Paget-Bailly et al. 2012; Stell and McGill 1973). Red meat is a risk factor for throat cancer, and Chinese medicine also believes that red meat can induce the disease (Di Maso et al. 2013). The synergistic action of free radicals and external risk factors in chronic inflammation may be one of the mechanisms of laryngeal cancer. Repeated direct stimulation of the laryngeal mucosa to generate tissue damage and repair stages of frequent alternating laryngeal reflux and gastric reflux, for example, could be a crucial endogenous cofactor in the pathogenesis of laryngeal cancer (Galli et al. 2006). HPV infection primarily promotes cancer occurrence by integrating the host cell genome, increasing proto-oncogene expression, and suppressing tumor suppressor gene expression. When HPV infects the larynx, for example, it can cause overexpression of the proto-oncogene Ras-like protein (*RAS*) or gene mutation, as well as failure of cell membrane signal transduction mediated by protein product *P21*, which can lead to malignant cell proliferation and laryngeal cancer (Yang et al. 2019a).

Modern medicine is mostly concerned with surgical resection in conjunction with radiotherapy and chemotherapy (Obid et al. 2019). Surgery, radiotherapy, and chemotherapy disrupt the human body’s healthy energy, resulting in a variety of adverse reactions such as dysphagia (Brisson-McKenna et al. 2023), poor language, impaired sense of smell and taste, and sputum production (Bui et al. 2018), as well as complications such as pharyngeal stenosis (Cui et al. 2018), pharyngeal fistula (Soliz et al. 2018), pharyngeal abscess, and permanent tracheostomy. Furthermore, due to the involvement of laryngeal precancer, the postoperative recurrence rate can be as high as 31.47% (Silver et al. 2009). Recurrence and metastasis are unavoidable.

A substantial body of medical research has demonstrated that traditional Chinese medicine can successfully ameliorate the adverse effects of anticancer and laryngeal cancer (So et al. 2019; Tan et al. 2020), decrease postoperative recurrence and metastasis, and improve patients’ quality of life (Xuewei et al. 2022). Bruceae Fructus is originally published in the Compendium of Materia Medica: “Bruceae Fructus, Fujian, Guangdong.” It can be bought in a variety of medical stores. It resembles a wu seed and has an oil-rich kernel. As cream, raw food makes you puke. It is the fruit of the Lamiaceae family’s Bruceae Fructus and is used to treat diarrhea, malaria, and cancer, among other things (Yan et al. 2017). Fructus brucellae fructus oil, Bruceae Fructus anti-tumor oil emulsion injection (Chen et al. 2022; Wang et al. 2021), and other traditional Chinese medicine products containing Bruceae Fructus as a raw material have been widely used in the clinical treatment of laryngeal cancer, nasopharyngeal cancer, and other malignant tumors and have been shown to reduce adverse reactions. The precise pharmacological action of Bruceae Fructus against laryngeal cancer is unknown.

With the modernization of traditional Chinese medicine, the updating and enhancement of important databases, and the ongoing maturation of technical means, network pharmacology has risen rapidly and is widely employed in numerous domains of traditional Chinese medicine (Fang et al. 2018). It has played a significant role in breaking through the obstacles of pharmacology and the precise mechanism of action of traditional Chinese medicine, the multi-component of traditional Chinese medicine compounds, and the multi-target of action. The gene expression omnibus (GEO) database is a tertiary resource for storing and retrieving publicly available high-throughput gene expression and genomic hybridization data (Edgar et al. 2002). When combined with network pharmacology, the weakness in network pharmacology caused by the limitation of the illness database is supplemented, and the bias induced by various forms of target action is decreased.

**Fig. 1:**
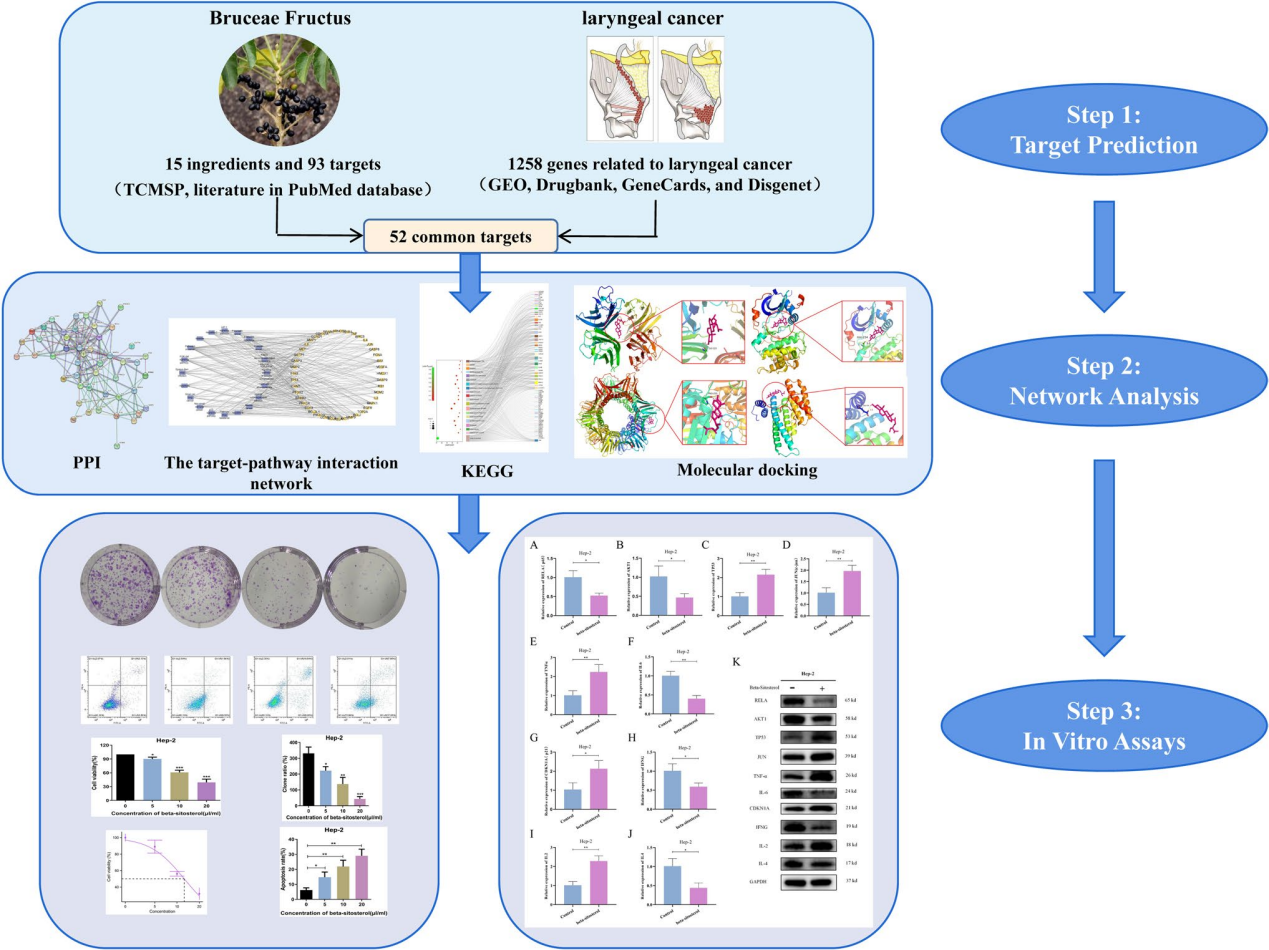
The detailed flow chart of the current study. This study integrated GEO, network pharmacology, and in vitro assays investigated the probable mechanism of Bruceae Fructus’ anti-laryngeal cancer activity

Based on the findings of the preceding studies, the specific mechanism of Brucea Fructus against laryngeal cancer was clarified, and a GEO gene chip combined with network pharmacology was proposed to investigate the pharmacological mechanism of Bruceae Fructus against laryngeal cancer, providing theoretical support for future research. The detailed flow chart of this study is shown in Fig. 1.

## Materials and methods

### Materials

Database: DrugBank database (https://go.drugbank.com), GeneCards database (https://www.disgenet.org), DisGeNET database (https://www.disgenet.org), GEO database (https://www.ncbi.nlm.nih.gov/geo), Traditional Chinese medicine systems pharmacology database and analysis platform (TCMSP) (https://old.tcmsp-e.com/tcmsp.php), UniProt Database (https://www.uniprot.org/), String database (https://cn.string-db.org/), the annotation, visualization and integrated discovery database (DAVID) (https://david-d.ncifcrf.gov), Bioinformatics (https://academic.oup.com/bioinformatics/?login=true), Venny (Version2.1.0, https://bioinfogp.cnb.csic.es/tools/venny), Protein Data Bank (PDB) database (https:/:/www.rcsb.org); software: R (https://www.r-project.org), Limma (Linear Models for Microarray Data), Cytoscape software (version 3.8), AutoDock software (version 4.2.6), AutoDock Tools software (version 1.5.6), PyMOL software (version 2.5.7).

### Screening of differential genes in laryngeal cancer

Laryngeal chips *GSE51985* were retrieved from the GEO database in MIAME Notation in Markup Language (MINiML) data format using the search term “laryngeal cancer.” The limma package of the R programming language (version 3.40.2) was used to investigate the differential expression of messenger ribonucleic acid (mRNA). In GEO, adjusted *P*-values were examined to eliminate false positive findings. Screen for differential expression of threshold mRNA was described as “Adjusted < 0.05 with log2(multiple changes) > 1 or log2(multiple changes) <  − 1.” After gathering the differentially expressed genes (DEGs) associated with laryngeal cancer, a volcano map and heat map were created.

### Laryngeal cancer disease gene acquisition and screening

The phrase “laryngeal cancer” was used to search the Drugbank, GeneCards, and DisGeNET databases for illness genes connected to laryngeal cancer. Disease genes from the Drugbank database, disease genes from the GeneCards database, and illness genes from the DisGeNET database were screened with a median “Relevance score” and “Score_gda > 0.1.” The genes related to laryngeal cancer were produced by integrating the genes obtained from the above database with the differential genes obtained from the *GSE51985* chip and deleting the duplicate genes.

### Screening of core components of Bruceae Fructus

The TCMSP database was used to retrieve the chemical components of Bruceae Fructus, and the active components were obtained under the toxicity pharmacokinetic of adsorption, distribution, metabolism, and excretion (ADME) standard, which met the two conditions of oral availability (OB) ≥ 30% and drug-likeness (DL) ≥ 0.18. After the active ingredients of each drug were obtained, the corresponding targets were also searched in the TCMSP database. Besides, the components and targets were supplemented in combination with literatures in the PubMed database. After merging, duplicate data were removed, and the UniProt database was used to standardize the acquired targets. Changed into a genetic symbol. Cytoscape software was used to create the active component-target network diagram of Bruceae Fructus, and the network analyzer feature was used to do topological analysis. The core components of Bruceae Fructus were screened based on “degree.”

### Construction of PPI networks and screening of core targets

The interaction targets of Bruceae Fructus and laryngeal cancer were obtained through Venny, and then the Venn diagram was drawn. A String database was used to create a protein–protein interaction (PPI) network between interacting targets. The mode was “Multiple Protein,” and the organism was “Homo sapiens.” The needed minimum interaction score was set to “maximum confidence (0.900)” to mask network disconnects. Other parameters stay constant. For visual analysis, the model, node2, and composite scores were loaded into Cytoscape software. Topology analysis was performed using the software’s network analyzer tool, and core-PPI-network and core network genes were screened using Matthews correlation coefficient (MCC) criteria. Then, the core targets of Bruceae Fructus treatment for laryngeal cancer were obtained.

### GO enrichment and KEGG pathway analysis.

The common targets of drugs and diseases were imported into the DAVID database, and the gene ontology (GO) and Kyoto encyclopedia of genes and genomes (KEGG) signaling pathway enrichment analysis (*P* < 0.05) was done using R software, and visualization plots were created. To confirm the possible functionality of the potential targets, the data was evaluated using feature enrichment. “OFFICIAL GENE SYMBOL” was selected as the identification, and “Homo sapiens” was selected as the species and background. Cell components (CC), molecular functions (MF), biological processes (BP), and KEGG pathways were analyzed. Data was sorted by *P*-value after exporting it. The 20 items with the lowest *P*-value for each GO enrichment and KEGG pathway were chosen, and advanced bubble maps were created using Bioinformatics. The ClusterProfiler tool in R is used to examine the GO function of possible mRNAs and enhance the KEGG pathway to better understand the carcinogenic significance of target genes. The R software package ggord was used to create the principal component analysis (PCA) graphic. The R software package heatmap was used to display the expression heatmap.

### Construct targets-pathways interaction network

The 20 KEGG pathway items with the lowest *P*-value were imported into Cytoscape software, and the targets of the interaction between substances in the KEGG pathway and illness targets were loaded into the software to build the targets-pathways interaction network.

### Molecular docking

The chosen core component and the core target were docked using the AutoDock method. The mol2 formats of the core components were downloaded from the TCMSP database, and PyMOL was used to transform them into pdb formats, then AutoDock Tools was used to save them as pdbqt formats. The pdb formats of the core targets were downloaded from the PDB database (protein complex with ligand and resolution of < 3A). PyMOL was used to delete water, add hydrogens, and remove the original ligand. The atomic type was set to Assign AD4 and then imported into AutoDockTools and saved in pdbqt format. The Lamarkian was chosen for AutoDock molecular docking, and the active pocket was defined as the location of the major ligand inside the protein complex. The binding free energy was used to screen the best docking outcomes, and the results were visualized by PyMOL.

### In vitro assays

#### Cell lines and drugs

The Hep-2 cells were obtained from FuHeng Biology (Shanghai, CHN) and validated by short tandem repeat (STR) analysis before being cultivated in Dulbecco’s modified Eagle’s medium (RPMI-1640, Hyclone, USA) containing 10% fetal bovine serum (FBS; BI, Israel). In a constant temperature incubator with 5% CO2, all cells were incubated in a full medium. MedChemExpress (MCE, https://www.medchemexpress.cn/bacoside-a. html, NO: HY-N0131) supplied the beta-sitosterol. The beta-sitosterol was kept at 4 °C and dissolved in 100 mg/mL stock solution of dimethyl sulfoxide (DMSO). The DMSO group with a level of less than 1/1000 was represented by the 0 μg/mL group.

#### Cell viability assay

Mahalanobis Taguchi system (MTS) tests were done on laryngeal cancer cells using a Promega Kit (Madison, WI, USA) according to the manufacturer’s instructions. In brief, 3000 Hep-2 cells were seeded onto 96-well plates at 100 L/well and grown under various treatment settings. Following the required time, 10 L of MTS solution was added to 90 L of RPMI-1640 each well, and the plates were incubated for 30 min. Following that, the absorbance of each well was measured at 490 nm using a microplate reader (Bio-Rad, USA).

#### Clone formation assay

Hep-2 cells were planted at 500 cells/well in 6-well plates to measure logarithmic growth. After cells adhered to the plate, they were either left untreated (control group, 0 μg/mL) or treated for 14 days with 5, 10, or 20 μg/mL beta-sitosterol. The cells were then fixed with 4% paraformaldehyde and stained with crystal violet staining solution. A high-definition (HD) camera was used to image stained colonies, and colonies with more than 50 cells were counted using Image J.

#### Flow cytometric assay

The Annexin V-FITC/PI Apoptosis Revelation rags (Beyotime, Shanghai, China) was hand-me-down to study cubicle apoptosis. In a nutshell, Hep-2 cells were virtuous adjacent to shorn phosphate-buffered vigor join epoch and resuspended. Soiling was accomplished according to the associate provided by the reagent shopkeeper, and able-bodied, make known cytometry (Beckman Coulter, Atlanta, GA, USA) was trick overseas coldness to scent apoptosis.

#### RNA extraction and RT-qPCR

RT-qPCR was used to detect mRNA expression levels of 10 key genes in Hep-2 cells treated with beta-sitosterol for 48 h. In short, total RNA was extracted from Hep-2 cells using the TRIzol reagent (TAKARA, 9109, Japan) according to the reagent supplier’s instructions. The Primrip TMRT kit (TAKARA, RR047A, Japan) was then used to synthesize complementary deoxyribonucleic acid (cDNA) from 1000 ng total RNA. Finally, TB Green®Premix Ex Taq™II (TAKARA, RR820A, Japan) was used as the standard control, and mRNA expression levels of 10 key genes were detected by RT-qPCR. Primer sequences of all genes are shown in Table 1.

**Table 1:** Primer-related information of 10 key genes and control genes

Primer name	Primer sequence	Species
RELA	5′-ACCCCTTCCAAGAAGAGCAG-3′	Human
	3′-TCACTCGGCAGATCTTGAGC-5′	
AKT1	5′-ACTGTCATCGAACGCACCTT-3′	Human
	3′-CTCCTCCTCCTCCTGCTTCT-5′	
TP53	5′-TGTGACTTGCACGTACTCCC-3′	Human
	3′-ACCATCGCTATCTGAGCAGC-5′	
JUN	5′-GCGGACCTTATGGCTACAGT-3′	Human
	3′-CCCGTTGCTGGACTGGATTA-5′	
TNF-α	5′-GCCCATGTTGTAGCAAACCC-3′	Human
	3′-TGAGGTACAGGCCCTCTGAT-5′	
IL-6	5′-TGCAATAACCACCCCTGACC-3′	Human
	3′-ATTTGCCGAAGAGCCCTCAG-5′	
CDKN1A	5′-GCGACTGTGATGCGCTAATG-3′	Human
	3′-GAAGGTAGAGCTTGGGCAGG-5′	
IFNG	5′-GTCGCCAGCAGCTAAAACAG-3′	Human
	3′-ACTGGGATGCTCTTCGACCT-5′	
IL-2	5′-ACAGGATGCAACTCCTGTCT-3′	Human
	3′-GCATCCTGGTGAGTTTGGGA-5′	
IL-4	5′-CTTTGCTGCCTCCAAGAACA-3′	Human
	3′-GTTCCTGTCGAGCCGTTTCA-5′	
GAPDH	5′-GGAGCGAGATCCCTCCAAAAT-3′	Human
	3′-GGCTGTTGTCATACTTCTCATGG-5′	

#### Western blot assay

The total protein of Hep-2 cells was extracted from RIPA lysate and its concentration was determined by BCA method. The protein concentration of 6% and 12% SDS-PAGE was increased to 4 μg/μL. The protein was deposited on the PVDF membrane after electrophoresis. The protein was then soaked in TBST for 5 min, rinsed, and sealed at room temperature with a quick-seal solution for 15 min. Then, used anti-RELA (1:1000), anti-AKT1 (1:1000), anti-TP53 (1:1000), anti-Jun (1:1000), anti-TNF-α (1:1000), anti-IL-6 (1:1000), anti-CDKN1A (1:1000), anti-ifng (1:1000), anti-IL-2 (1:1000), anti-IL-4 (1:1000), and anti-GAPDH (1:1000) shut down the system overnight at 4 °C. The primary antibody was washed; the secondary antibody labeled with horseradish peroxidase (1:2000) was added, incubated at room temperature for 1 h, and then TBST rinsed 3 times for 5 min. Excess liquid on the film is absorbed with filter paper and then developed in the dark with ultra-sensitive ECL. Finally, using GAPDH as internal control, the relative expression levels of 10 key genes were measured by scanning protein bands.

## Results

### Acquisition of laryngeal oncogenes

**Fig. 2:**
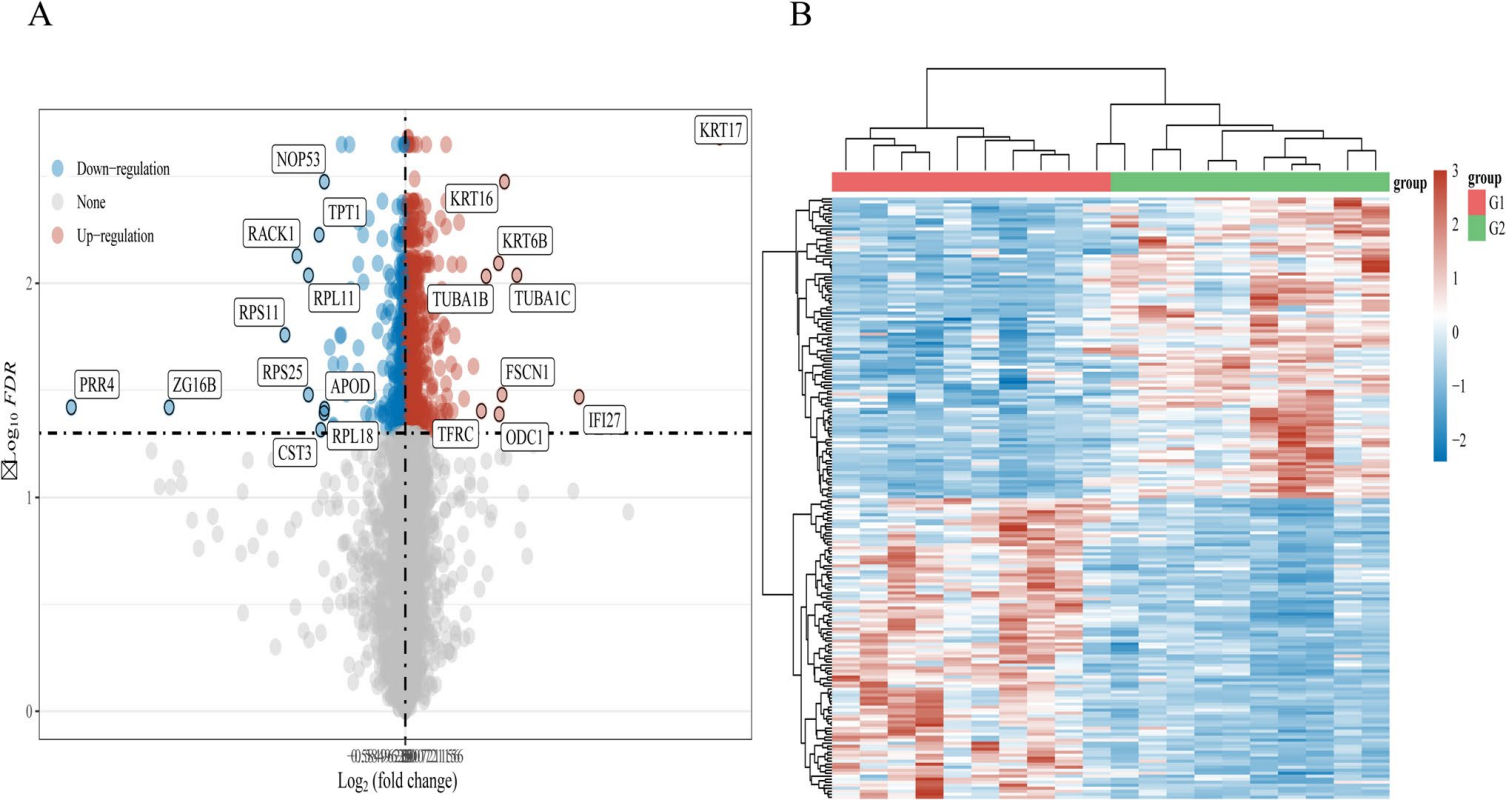
**A** DEGs volcano map (note: blue represented down-regulation and red represented up-regulation). **B** Heat map (note: the red area in the first row corresponded to G1 and the green area corresponds to G2. In heat map, blue represented down-regulation and red represents up-regulation)

The *GSE51985* chip was retrieved from the GEO database, and the differential expression of mRNA was examined and screened using R software. A volcano map and heat map of the DEGs were created using the 1272 different genes from laryngeal cancer (Fig. 2A, B).

The search term “laryngeal cancer” was used to look for and evaluate disease targets associated with laryngeal cancer in the Drugbank, GeneCards, and DisGeNET databases. One thousand two hundred fifty-eight genes associated with laryngeal cancer were produced by merging laryngeal cancer genes found in the aforementioned database and eliminating duplicate genes.

### Bruceae Fructus composition and target acquisition

**Fig. 3:**
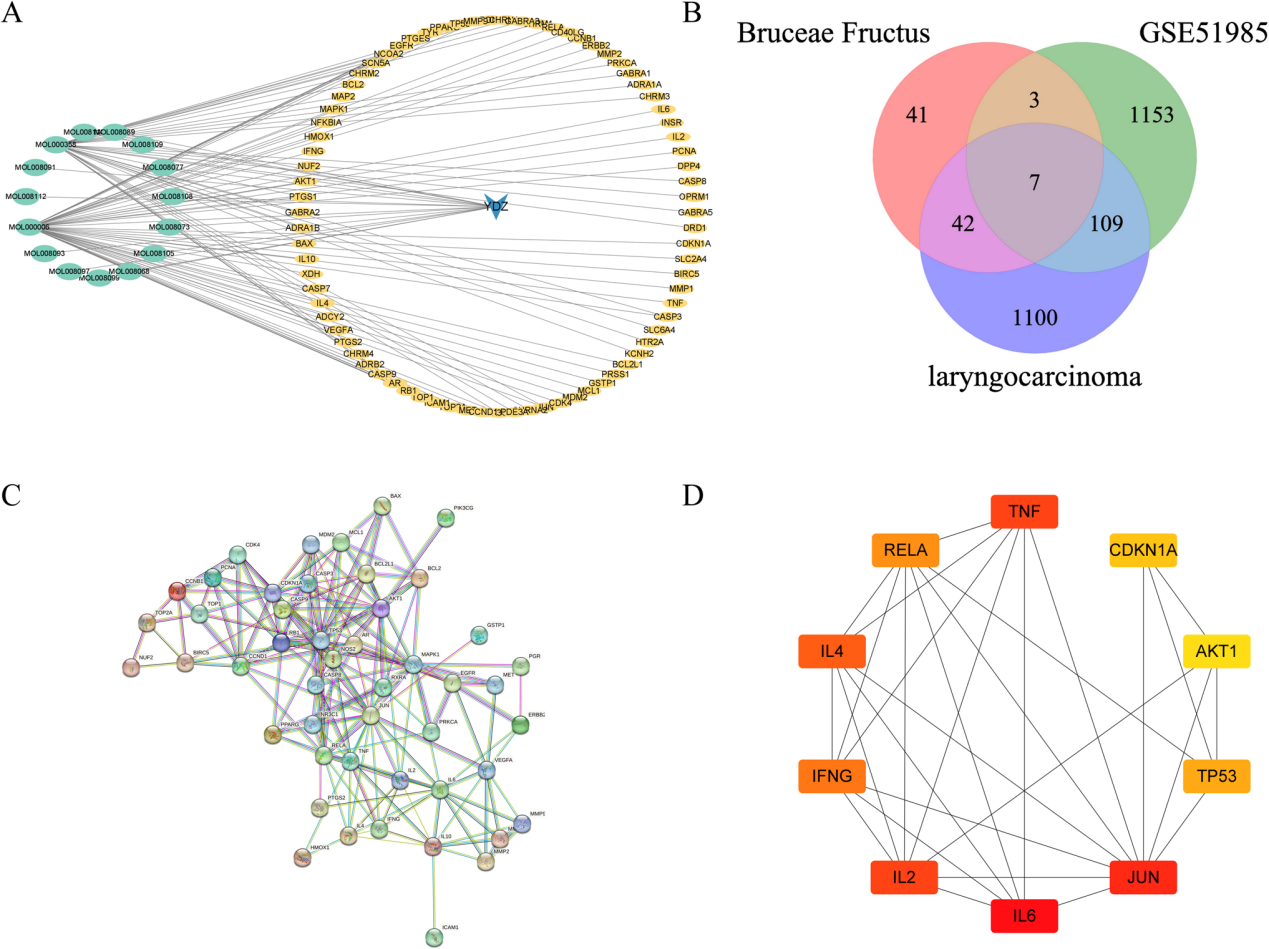
**A** Active ingredients-targets interaction network diagram of Bruceae Fructus (note: the blue arrows represented Bruceae Fructus, green ovals represented the active ingredients, and yellow squares represented the targets). **B** Venn diagram. There were 52 intersection targets of Bruceae Fructus and laryngeal cancer). **C** PPI network. The nodes disconnected from the network were hidden. **D** MCC. The core targets of the treatment of laryngeal cancer were selected as *IL6*, *JUN*, *TNF*, *IL2*, *IL4*, *IFNG*, *RELA, TP53*, *CDKN1A*, *AKT1*

The components for Bruceae Fructus were collected from the TCMSP database, and 15 useful components were obtained after the non-target components were eliminated using the criteria of OB ≥ 30% and DL ≥ 0.18. After merging targets in the TCMSP database and literatures in the PubMed database, duplicate data were removed, and 93 targets were found utilizing the Uniprot database for standardized processing. The component-target interaction network for Bruceae Fructus was mapped using Cytoscape software (Fig. 3A). Beta-sitosterol and luteolin were the main ingredients of Bruceae Fructus, according to the function value of the software’s network analyzer.

### Construction of PPI networks and screening of core targets

Venny was used to get 52 intersection targets of Bruceae Fructus and laryngeal cancer (Fig. 3B), and each intersection target was loaded into the String database to build a PPI network (the nodes disconnected from the network were hidden) (Fig. 3C). Other parameters stay constant. For visual analysis, the model, node2, and composite scores were loaded into Cytoscape software. Topological analysis was performed by using the network analyzer function of the software, and the core targets of the treatment of laryngeal cancer were selected as interleukin-6 (*IL6*), transcription factor *AP-1* (*JUN*), tumor necrosis factor (*TNF*), interleukin-2 (*IL2*), interleukin-4 (*IL4*), interferon gamma (*IFNG*), *RELA* proto-oncogene (*RELA*), tumor protein *P53* (*TP53*), cyclin-dependent kinase inhibitor 1A (*CDKN1A*), and serine/threonine kinase 1 (*AKT1*) according to the MCC value (Fig. 3D).

### GO enrichment and KEGG pathway analysis

The Bruceae Fructus and laryngeal cancer intersection targets were imported into the DAVID database for GO enrichment and KEGG pathway analysis. There were 41 CC, 78 MF, 383 BP, and 108 KEGG pathways identified. The data was exported and sorted by the *P*-value. For each GO enrichment and KEGG pathway, the 20 item days with the lowest *P*-value were chosen, and bioinformatics created advanced bubble maps. The deeper the bubble color, the lower the *P*-value, the larger the bubble, and the more genes there were.

**Fig. 4:**
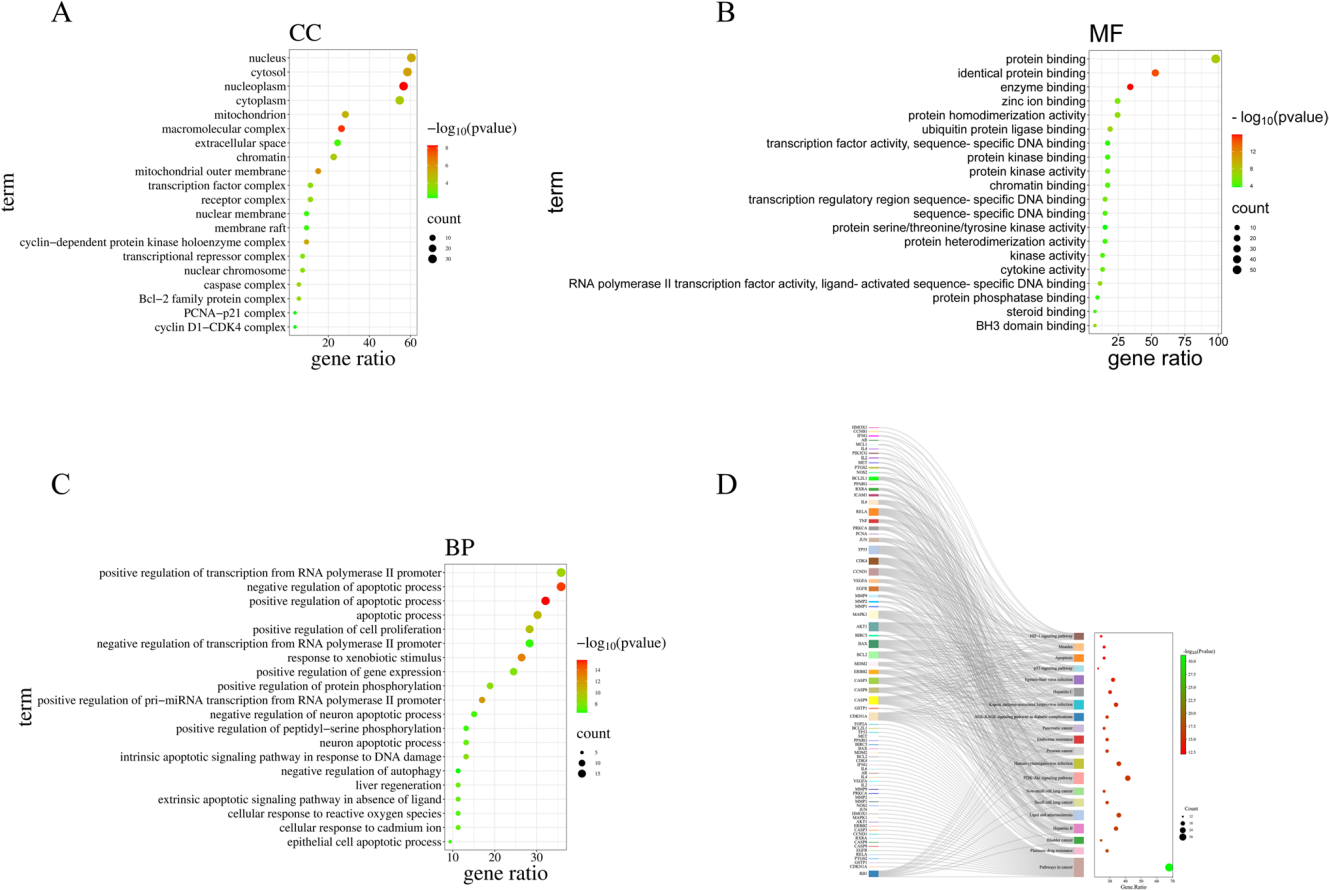
**A** Bubble diagram of GO-CC. **B** Bubble diagram of GO-MF. **C** Bubble diagram of GO-BP. **D** Bubble diagram of KEGG (note: the larger the circle in the figure, the larger the number of relevant targets)

The top 20 results of GO-CC enrichment were as follows: nucleoplasm, macromolecular complex, mitochondrial outer membrane, cytosol, cyclin-dependent protein kinase holoenzyme complex, nucleus, mitochondrion, cytoplasm, chromatin, caspase complex, Bcl-2 family protein complex, receptor complex, transcription factor complex, nuclear chromosome, transcriptional repressor complex, extracellular space, membrane raft, nuclear membrane, cyclin D1-cyclin-dependent kinase 4 (*D1-CDK4*) complex, proliferating cell nuclear antigen (*PCNA*)-*P21* complex (Fig. 4A).

The top 20 results of GO-MF enrichment results were enzyme binding, identical protein binding, protein binding, ubiquitin protein ligase binding, ribonucleic acid (RNA) polymerase II transcription factor activity, ligand-activated sequence-specific deoxyribonucleic acid (DNA) binding, *BH3* domain binding, protein homodimerization activity, zinc ion binding, transcription regulatory region sequence-specific DNA binding, protein kinase activity, cytokine activity, kinase activity, chromatin binding, sequence-specific DNA binding, protein heterodimerization activity, steroid binding, protein kinase binding, protein phosphatase binding, transcription factor activity, sequence-specific DNA binding, and protein serine/threonine/tyrosine kinase activity (Fig. 4B).

The top 20 results of GO-BP enrichment were positive regulation of the apoptotic process, negative regulation of the apoptotic process, response to xenobiotic stimulus, positive regulation of pri-miRNA transcription from RNA polymerase II promoter, apoptotic process, positive regulation of cell proliferation, positive regulation of transcription from RNA polymerase II promoter, intrinsic apoptotic signaling pathway in response to DNA damage, positive regulation of protein phosphorylation, positive regulation of gene expression, liver regeneration, extrinsic apoptotic signaling pathway in absence of ligand, cellular response to cadmium ion, neuron apoptotic process, cellular response to reactive oxygen species, negative regulation of neuron apoptotic process, positive regulation of peptidyl-serine phosphorylation, epithelial cell apoptotic process, negative regulation of transcription from RNA polymerase II promoter, and negative regulation of autophagy (Fig. 4C).

**Fig. 5:**
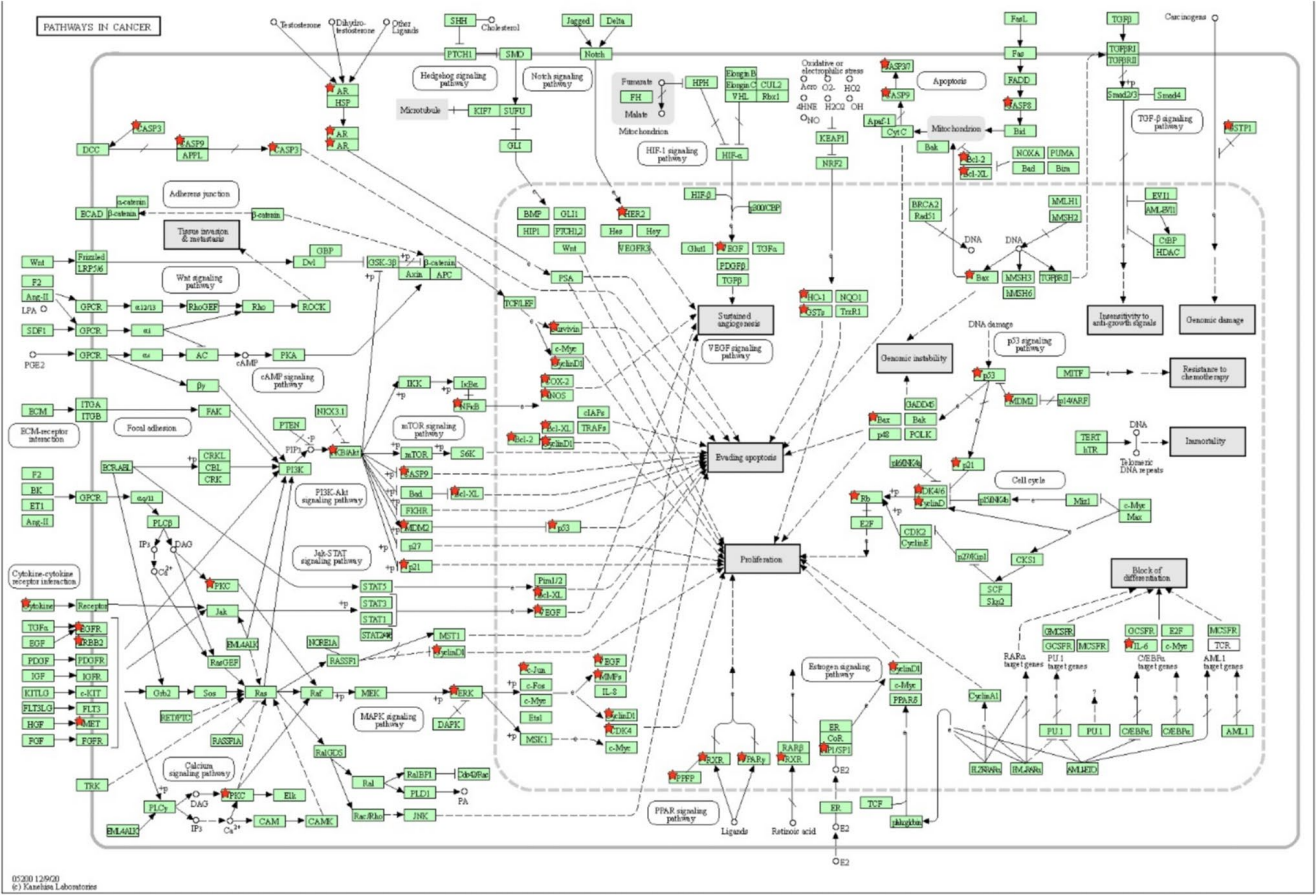
Pathways in cancer (note: red pentagons were the interaction targets of Bruceae Fructus and laryngeal cancer contained in the pathways in cancer)

The top 20 KEGG concentration results were pathways in cancer, platinum drug resistance, bladder cancer, hepatitis B, lipid and atherosclerosis, small cell lung cancer, non-small cell lung cancer, phosphatidyl inositol 3 kinases (*PI3K*)-serine/threonine kinase (*Akt*) signaling pathway, human cytomegalovirus infection, prostate cancer, endocrine resistance, pancreatic cancer, advanced glycation end product (*AGE*)-receptor for advanced glycation end products (*RAGE*) signaling pathway in diabetic complications, Kaposi sarcoma–associated herpesvirus infection, hepatitis C, Epstein-Barr virus infection, *P53* signaling pathway, apoptosis, measles, and hypoxia-induced factor-1 (*HIF-1*) signaling pathway (Fig. 4D). Pathways in cancer are shown in the figure below (Fig. 5).

### Construct targets-pathways interaction network

**Fig. 6:**
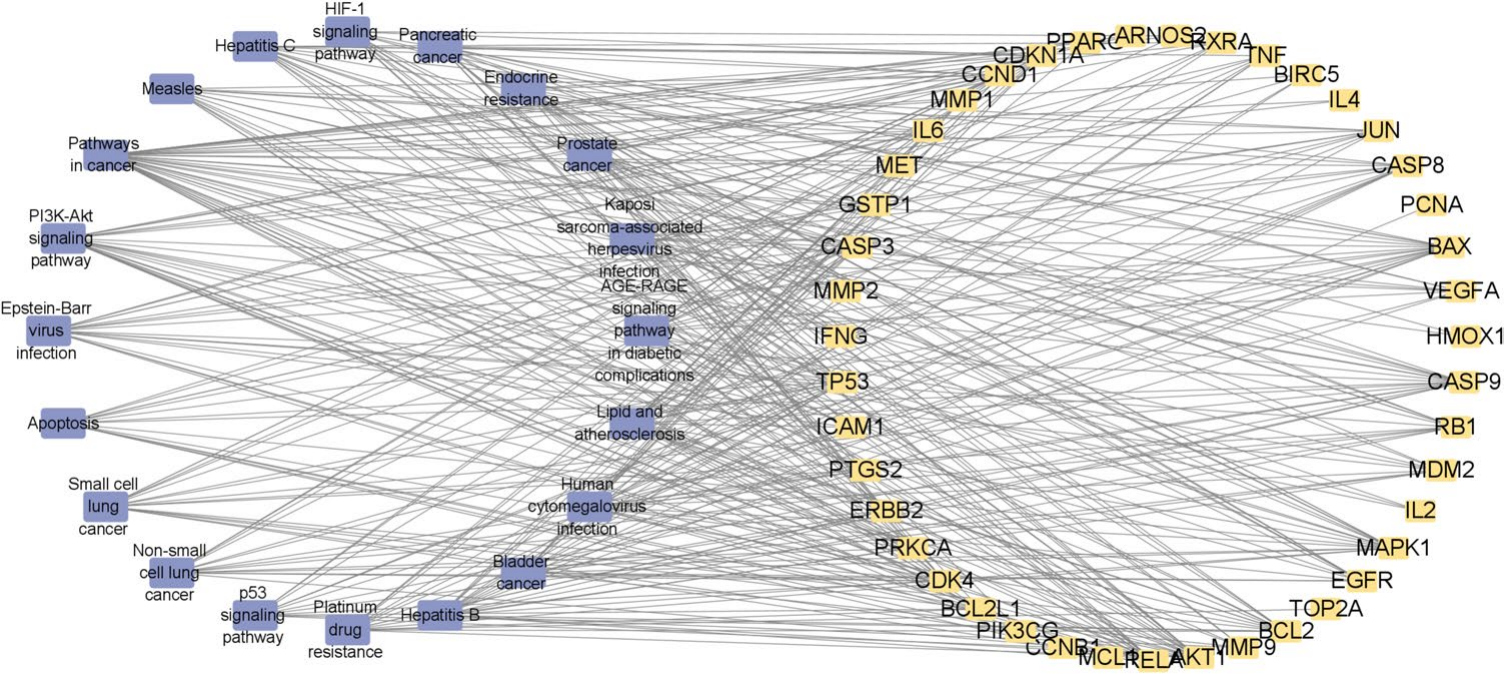
The targets-pathways interaction network (note: Yellow squares represented the targets, and bluish violet squares represented the pathways)

The targets-pathways interaction network was constructed by Cytoscape software (Fig. 6). It contains 20 signaling pathways and 43 related genes. Yellow squares represent the targets, and bluish violet squares represent the pathways.

### Molecular docking results

**Fig. 7:**
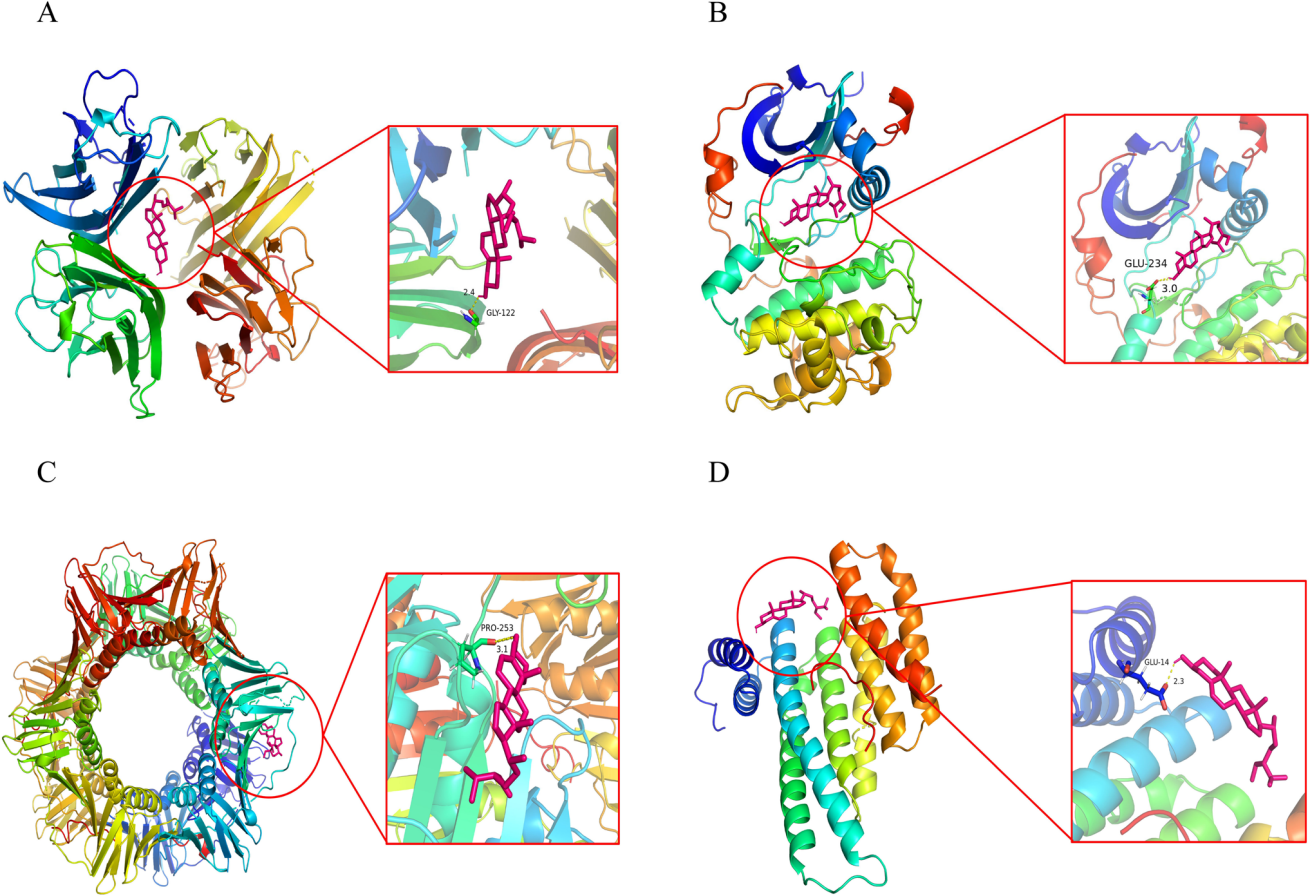
Molecular docking results. **A** The docking mode of *TNF* and beta-sitosterol. **B** The docking mode of *AKT1* and beta-sitosterol. **C** The docking mode of *CDKN1A* and beta-sitosterol. **D** The docking mode of *TP53* and beta-sitosterol

Molecular docking was carried out between the core components (beta-sitosterol and luteolin) and the core targets (*IL6*, *JUN*, *TNF*, *IL2*, *IL4*, *IFNG*, *RELA*, *TP53*, *CDKN1A*, *AKT1*). The lower the binding free energy between the ligand and the receptor, the more stable the conformation, indicating a better affinity. When the binding free energy is less than zero, the ligand and receptor can bond spontaneously. The binding energy results obtained by molecular docking in this study were shown (Table 2). The molecular docking results with the lowest binding free energy were visualized by PyMOL (Fig. 7A–D).

**Table 2:** Molecular docking results

Compounds	Target	PDB ID	Grid box (X, Y, Z)	Binding free energy (kcal/mol)
Beta-sitosterol	IL6	1ALU	(− 7.677, − 12.743, 0.048)	− 3.57
	JUN	5FV8	(34.364, 3.119, − 3.095)	− 5.34
	TNF	2AZ5	(− 19.163, 74.452, 33.837)	− 8.85
	IL2	4NEM	(− 14.928, 8.061, 18.532)	− 4.75
	IL4	4YDY	(− 21.665, 4.809, 26.921)	− 5.05
	IFNG	3BES	(46.236, 18.985, 20.464)	− 5.51
	RELA	6NV2	(18.797, 20.287, − 1.21)	− 3.56
	TP53	6R5L	(18.11, 20.174, − 4.767)	− 6.47
	CDKN1A	6CBI	(23.204, − 20.841, 166.111)	− 6.6
	AKT1	6CCY	(− 9.801, 15.312, − 31.398)	− 8.0
Luteolin	IL6	1ALU	(− 7.677, − 12.743, 0.048)	− 3.18
	JUN	5FV8	(34.364, 3.119, − 3.095)	− 4.22
	TNF	2AZ5	(− 19.163, 74.452, 33.837)	− 5.32
	IL2	4NEM	(− 14.928, 8.061, 18.532)	− 3.94
	IL4	4YDY	(− 21.665, 4.809, 26.921)	− 4.1
	IFNG	3BES	(46.236, 18.985, 20.464)	− 4.74
	RELA	6NV2	(18.797, 20.287, − 1.21)	− 3.73
	TP53	6R5L	(18.11, 20.174, − 4.767)	− 4.55
	CDKN1A	6CBI	(23.204, − 20.841, 166.111)	− 4.7
	AKT1	6CCY	(− 9.801, 15.312, − 31.398)	− 6.2

### The results of in vitro assays

#### Beta-sitosterol inhibits the proliferation of human laryngeal cancer cells

**Fig. 8:**
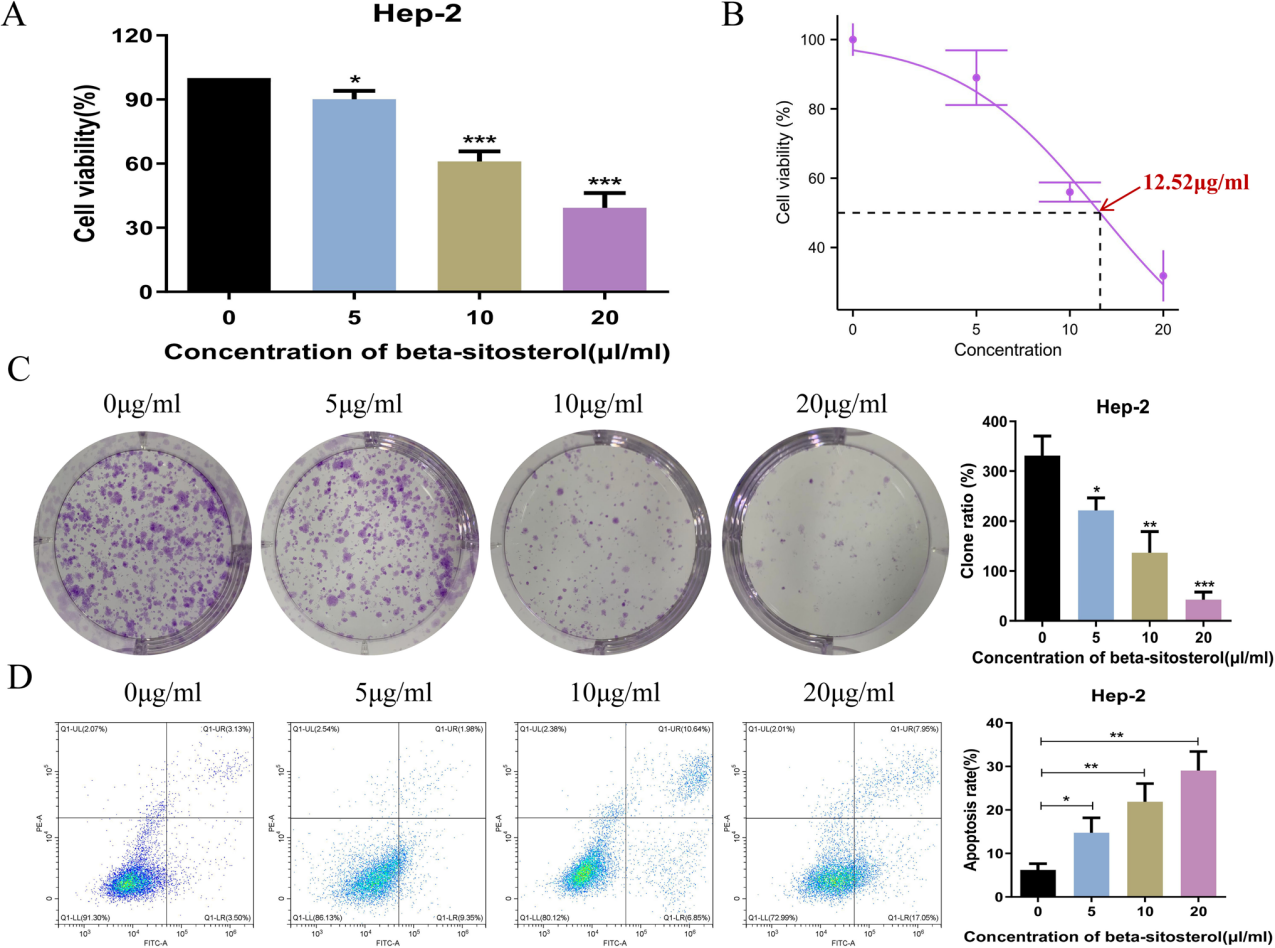
The results of in vitro assays. **A** Beta-sitosterol suppressed Hep-2 cell activity in a concentration-dependent manner. **B** At 48 h, the median inhibitory concentration (IC50) was 12.57 μg/mL. **C** Treatment with a low quantity of beta-sitosterol somewhat reduced colony formation compared to the control (0 μg/mL beta-sitosterol), but treatment with a high concentration of beta-sitosterol greatly reduced colony formation. **D** The effect of beta-sitosterol on laryngeal cancer cell apoptosis

To explore the biological effects of beta-sitosterol, the viability of laryngeal cancer cells were assessed after beta-sitosterol therapy using the MTS technique. The results revealed that beta-sitosterol suppressed Hep-2 cell activity in a concentration-dependent manner (Fig. 8A). At 48 h, the median inhibitory concentration (IC50) was 12.57 μg/mL (Fig. 8B). Following that, how beta-sitosterol affected colony formation after 14 days of culture of treated cells was researched. Treatment with a low quantity of beta-sitosterol somewhat reduced colony formation compared to the control (0 μg/mL beta-sitosterol), but treatment with a high concentration of beta-sitosterol greatly reduced colony formation (Fig. 8C). These findings revealed that beta-sitosterol has powerful antiproliferative properties in Hep-2 cells.

#### Beta-sitosterol promotes apoptosis of Hep-2 cells

To investigate the effect of beta-sitosterol on laryngeal cancer cell apoptosis, flow cytometry was used to detect apoptosis. As shown in Fig. 8D, beta-sitosterol promoted laryngeal cancer cell apoptosis in a concentration-dependent manner.

#### Validation of mRNA and protein expression levels of ten core genes

**Fig. 9:**
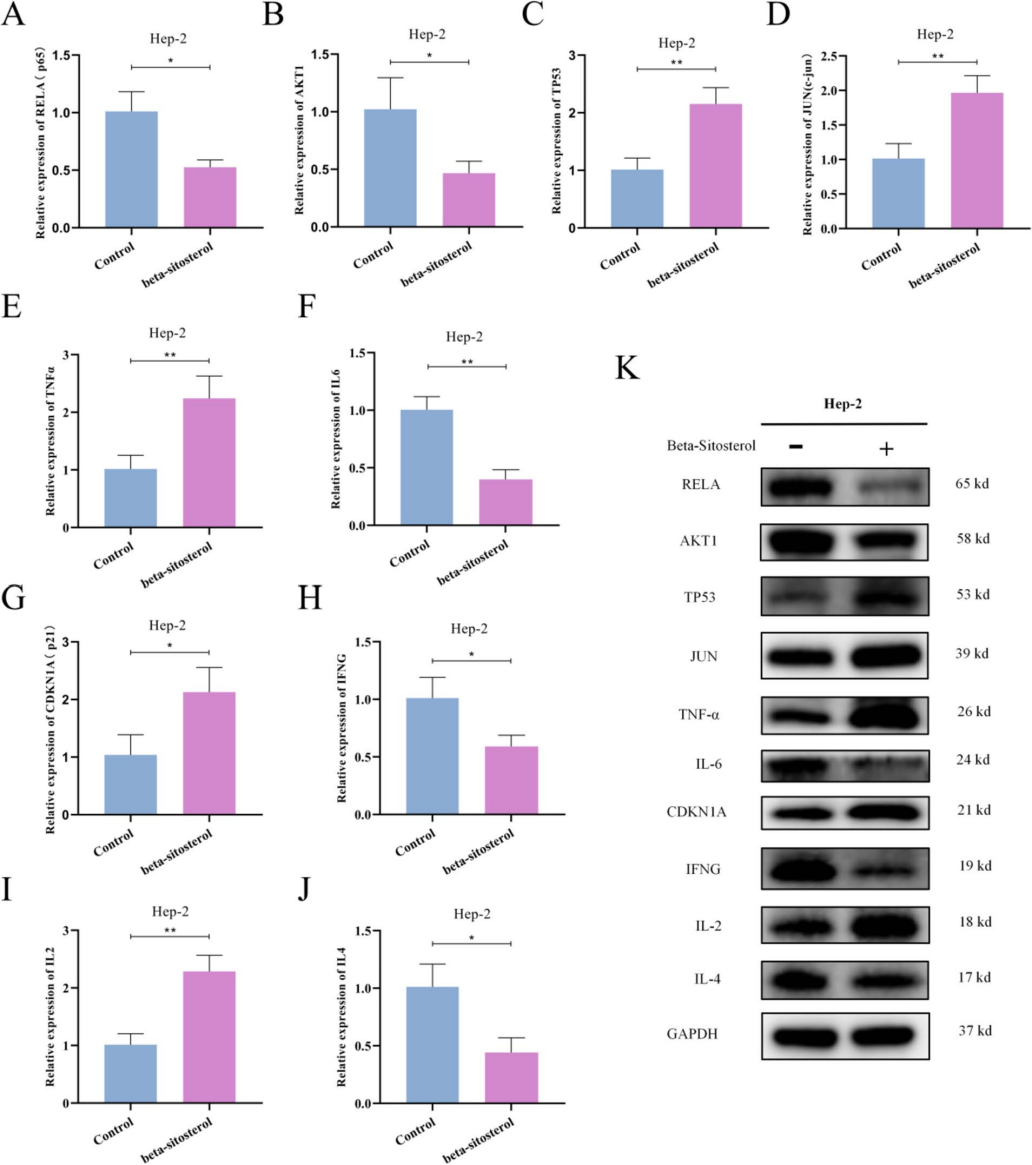
**A**–**J** mRNA expression levels of 10 key genes in Hep-2 cells treated with 12.57 μg/mL beta-sitosterol were detected by RT-qPCR. **K** Protein expression levels of 10 key genes in Hep-2 cells treated with beta-sitosterol were detected by Western Blot. *P < 0.05, **P < 0.01

In addition, to further demonstrate the reliability of our network pharmacological prediction, we treated Hep-2 cells with beta-sitosterol of IC50 with 12.57 μg/mL for 48 h, and then detected mRNA and protein expression levels of 10 key genes by RT-qPCR and Western Blot assay. The results showed that the mRNA and protein expression levels of TP53, JUN, TNF-α, CDKN1A, and IL-2 were significantly up-regulated after beta-sitosterol treatment, while the mRNA and protein expression levels of RELA, AKT1, IL-6, IFNG, and IL-4 were significantly down-regulated. This further proved to be consistent with the predicted results (Fig. 9A–K).

## Discussion

Bruceae Fructus includes beta-sitosterol, luteolin, bristol, and other components, with beta-sitosterol and luteolin being the most important. The anticancer mechanism is related to promoting apoptosis, affecting the cell cycle, anti-proliferation, anti-invasion, anti-angiogenesis, and anti-metastasis (Khan et al. 2022). Beta-sitosterol, a non-toxic white powdered organic substance, has anti-inflammatory, hepatoprotective, antioxidant, cardioprotective, and anti-diabetic effects. A fundamental anticancer technique is the removal of damaged or undesirable cells, known as apoptosis or programmed cell death. Different signaling pathways involving distinct regulatory proteins control apoptosis. To fight cancer, beta-sitosterol can disrupt several cell signaling pathways and either directly or indirectly cause apoptosis (Bao et al. 2022), such as activating *P53* (Rajavel et al. 2018), regulating the *PI7K/Akt/mTOR* pathway (Zhu et al. 2018), mediating the apoptotic regulator *Bcl-2* protein family (Moon et al. 2008; Vundru et al. 2013), and generating Bax and activating caspase (Choi et al. 2003). Wang L demonstrated that beta-sitosterol may suppress tubulin expression and the polymerization of intracellular microtubules in cell tests, implying that the anti-microtubule activity of beta-sitosterol could be a key factor for reducing cancer cell proliferation (Wang et al. 2006). Luteolin is a flavonoid molecule with anti-inflammatory, neuroprotective, antiallergic, and cancer-fighting properties. Luteolin’s anticancer activities include triggering apoptosis, reducing cell growth, decreasing metastasis and angiogenesis, and sensitizing different cancer cells. Induces cell death by oxidation–reduction (REDOX) control, DNA damage, and protein kinase suppression of cancer cell growth as well as metastasis and angiogenesis inhibition (Lin et al. 2008). Zhang H et al. demonstrated that luteolin not only activated caspase-2 and caspase-3 to induce apoptosis in Hep-8 cells, but also activated the receptor-level Fas signaling pathway in Hep-2 cells to produce apoptosis (Zhang et al. 2014).

The PPI network’s core targets in this study were *IL6*, *JUN*, *TNF*, *IL2*, *IL4*, *IFNG*, *RELA*, *TP53*, *CDKN1A*, and *AKT1*. Cytokines are components of the immune response, and there is a link between tumor growth and cytokine dysregulation. *IL6* is an inflammatory cytokine gene found in nearly all tumor microenvironments. Ibrahim postulated that inhibiting *IL6* may decrease tumor cell survival pathways, making tumor cells more susceptible to immunotherapy and chemotherapy (Ibrahim et al. 2020). *TNF* is a tumor necrosis factor gene, while *IL2* is a cytokine gene. Ulrike Stein confirmed that after transducing tumor cells, *IL2* and *TNF* modify treatment resistance (Stein et al. 1996). *IL4* is a gene that codes for a key T helper 2 (*Th2*) cytokine that promotes tumor growth by increasing proliferation and survival. Roca H confirmed that *IL4* activated the *JUN* N-terminal kinases (*JNK*) pathway and caused survivin-dependent proliferation of prostate cancer PC3 cells (Roca et al. 2012). *IFNG* is a major host response regulator of intracellular pathogen replication (Al-Zeer et al. 2013), as well as possessing antiviral, antibacterial, and anti-tumor properties (Miller et al. 2009). Mito I investigated *IFNG* and discovered that it is substantially expressed in lymphocytes from head and neck squamous cell cancer (HNSCC) (Mito et al. 2021). *JUN* is a proto-oncogene that activates gene expression to participate in cell proliferation, survival, and apoptosis. Que YH postulated that *JUN* gene overexpression and the c-Jun protein’s imbalance of cell proliferation and differentiation could contribute to laryngeal cancer (Que and Ma 2003). According to Shaulian E, the effect of jun protein on tumor transformation is connected to the environment. *JunB* proto-oncogene (*JunB*) has bidirectional regulation and can function as an oncogene or tumor suppressor (Shaulian 2010). Nuclear factor kappa-B cell (*NF-KB*) is a cellular molecule that can prevent cell death as well as sustain and promote cell development. Du J revealed that the *P65* protein produced by the *RELA* gene had a positive connection with viral protein *E7*, and nuclear localization of *P65* indicated that *NF-KB* was continually active in laryngeal cancer cells (Du et al. 2003). The *TP53* gene encodes the *P53* protein, which influences cell cycle and cell longevity through participating in genome repair and recombination, controlling cell metabolism, monitoring stress signals, and so on (Lane and Levine 2010). According to Zhou G, the *TP53* mutation rate in laryngeal cancer can reach 83.5%, and *TP53* mutations can alter the progression and efficacy of HNSCC (Zhou et al. 2016). *CDKN1A* is the gene that encodes the *P21* cyclin–dependent kinase inhibitor, which inhibits cell growth and activates the DNA damage response (Yimit et al. 2019). According to Chernock RD, patients with *CDKN1A* (*P21*) positive laryngeal cancer have a better prognosis (Chernock et al. 2013).

GO enrichment and KEGG pathway analysis showed that cancer pathways, platinum drug resistance, the *PI3K-Akt* signaling pathway, pancreatic cancer, the *P53* signaling pathway, apoptosis, the *HIF-1* signaling pathway, and so on are directly related to laryngeal cancer and are associated with deregulation of various pathways related to cell differentiation, cell cycle control, apoptosis, angiogenesis, and metastasis. In laryngeal cancer, the *PI3K-Akt* signaling pathway is a frequent oncogenic mutation route and a mutant mitotic pathway (Lui et al. 2013). For example, by stimulating the *PI3K-Akt* pathway, long non-coding RNA-growth-arrest-specific transcript 5 (*GAS5*) and Zanthoxanthus pepper seed oil can achieve anti-proliferation, anti-metastasis, or pro-apoptosis (Bai et al. 2021; Liu et al. 2021). Low oxygen levels can result from the growth of cancer cells and the erratic development of blood vessels, which is one of the major reasons why people die. It can control tumor angiogenesis, proliferation, invasion, metastasis, and immune system via activation of the *HIF-1* signaling pathway and modulation of hypoxia-inducing factor transcription (Mamlouk and Wielockx 2013). Several biological processes, including DNA repair, apoptosis, cell aging, and the cell cycle, are regulated by the *P53* signaling pathway. Yang J found that *DIAPH1* induces apoptosis via *ATR/P53/caspase 8/3* in laryngeal squamous cell cancer cells (Yang et al. 2019b). Xie Z was also involved in the stimulation of apoptosis via the *P53* signaling pathway in animal tests (Xie et al. 2022). Platinum medicines, which can serve as cytotoxic agents in cancer cells by destroying DNA and triggering apoptosis, are extensively employed in the treatment of several malignancies, including throat cancer, and their resistance is currently a major issue. Tian L proposed that Hep-2/R cisplatin resistance was caused by *ATF2* overexpression, and also demonstrated that miR-26b reversed Hep-2/R cisplatin resistance by reducing *ATF2* expression, hence increasing the therapeutic impact (Tian et al. 2017).

To investigate the biological effects of beta-sitosterol, we first assessed the viability of laryngeal cancer cells after beta-sitosterol therapy using the MTS technique. The findings demonstrated that beta-sitosterol inhibited Hep-2 cell activity in a concentration-dependent manner. Furthermore, in Hep-2 cells, beta-sitosterol shows potent antiproliferative effects. Flow cytometry was utilized to detect apoptosis to examine the influence of beta-sitosterol on laryngeal cancer cell apoptosis. The findings revealed that beta-sitosterol induced apoptosis in laryngeal cancer cells in a concentration-dependent way. The results of RT-qPCR and Western Blot assay showed that the mRNA and protein expression levels of *TP53*, *JUN*, *TNF-α*, *CDKN1A*, and *IL-2* were significantly up-regulated after beta-sitosterol treatment, while the mRNA and protein expression levels of *RELA*, *AKT1*, *IL-6*, *IFNG*, and *IL-4* were significantly down-regulated. This further proved to be consistent with the predicted results.

## Conclusion

This study demonstrated the multi-compound, multi-target, and multi-pathway anti-laryngeal cancer of Bruceae Fructus, and explained the probable mechanism associated to the anti-laryngeal cancer of Bruceae Fructus for the first time. Brucea Fructus has a vital function in anti-laryngeal cancer. *PI3K-Akt* signaling pathway, *P53* signaling pathway, and other signaling pathways may all play a role in platinum drug resistance reduction, promoting apoptosis, promoting DNA repair, anti-proliferation, anti-metastasis, regulating cell cycle, and regulating hypoxia. The results of the cell experiment showed that beta-sitosterol have antiproliferative effects, which can reduce Hep-2 cell activity and increase apoptosis in laryngeal cancer cells. The mRNA and protein expression levels of *TP53*, *JUN*, *TNF-α*, *CDKN1A*, and *IL-2* were significantly up-regulated after beta-sitosterol treatment, while the mRNA and protein expression levels of *RELA*, *AKT1*, *IL-6*, *IFNG*, and *IL-4* were significantly down-regulated. There are some limitations in our article, such as lack Determination of Compound Content by Liquid Chromatography, and that is what we are going to research at next. Although the precise mechanism of action of Bruceae Fructus requires further investigation, this study gives theoretical justification for future animal trials.

## Data Availability

The data that support the findings of this study are available from the corresponding author, upon reasonable request.

## Abbreviations

ADME: Adsorption, distribution, metabolism, and excretion
*AGE*: Advanced glycation end product
*AKT*: Serine/threonine kinase
*AKT1*: Serine/threonine kinase 1
*ATF2*: Activating transcription factor 2
*Bcl-2*: Apoptosis regulator Bcl-2
BP: Biological processes
CC: Closeness centrality
*CDKN1A*: Cyclin-dependent kinase inhibitor 1A
DAVID: The annotation, visualization, and integrated discovery database
DEGs: Discovering differentially expressed genes
DL: Drug-likeness
DMSO: Dimethyl sulfoxide
DNA: Deoxyribonucleic acid
*D1-CDK4*: D1-cyclin-dependent kinase 4
FBS: Fetal bovine serum
*GAS5*: Growth-arrest-specific transcript 5
GEO: Gene expression omnibus
GO: Gene ontology
HD: High definition
*HIF-1*: Hypoxia-induced factor-1
HPV: Human papilloma virus
IC50: The median inhibitory concentration
*IL2*: Interleukin-2
*IL4*: Interleukin-4
*IL6*: Interleukin-6
*IFNG*: Interferon Gamma
*JNK*: JUN N -terminal kinases
*JUN*: Transcription factor AP-1
*JUNB*: JunB proto-oncogene
KEGG: Kyoto encyclopedia of genes and genomes
MCC: Matthews correlation coefficient
MCE: MedChemExpress
MF: Molecular function
MINiML: MIAME notation in markup language
mTOR: Serine/threonine-protein kinase mTOR
mRNA: Messenger ribonucleic acid
*NF-kB*: Nuclear factor kappa-B cell
MTS: Mahalanobis Taguchi system
OB: Oral bioavailability
PAHs: Polycyclic aromatic hydrocarbons
PCA: Principal component analysis
*PCNA*: Proliferating cell nuclear antigen
PDB: Protein Data Bank
*PI3K*: Phosphatidyl inositol 3 kinases
*PI7K*: Phosphatidyl inositol 7 kinases
PPI: Protein-protein interaction
RAGE: Receptor for advanced glycation end products
*RAS*: Ras-like protein
REDOX: Oxidation-reduction
*RELA*: *RELA* Proto-oncogene
RNA: Ribonucleic acid
STR: Short tandem repeat
TCMSP: Traditional Chinese medicine systems pharmacology database and analysis platform
*Th2*: T helper 2
*TNF*: Tumor necrosis factor
*TP53*: Tumor protein *P53*
